# P-855. Epidemiology and Clinical Pattern of Bacteremia in a Referral Hospital in Nicaragua: a Three Year Cohort Study

**DOI:** 10.1093/ofid/ofae631.1047

**Published:** 2025-01-29

**Authors:** Anielka Solís-Altamirano, Karina Colomer-Sánchez, Kevin Gavarrete-Rivas, Guillermo D Porras-Cortés

**Affiliations:** Hospital Dr. Fernando Vélez Paiz, León, Leon, Nicaragua; Hospital Dr. Fernando Vélez Paiz, León, Leon, Nicaragua; Hospital Dr. Fernando Vélez Paiz, León, Leon, Nicaragua; Hospital Dr. Fernando Vélez Paiz, León, Leon, Nicaragua

## Abstract

**Background:**

In recent years, some institutions have described changes in the epidemiological, microbiological, and clinical profiles of bacteremias. The Dr. Fernando Vélez Paiz Hospital (HFVP) is a teaching and referral hospital in Nicaragua, which opened its services 5 years ago. The aim of this study is to describe and analyze the epidemiological, microbiological, and clinical characteristics as well as risk factors for mortality of patients with bacteremia.

**Table 1.**
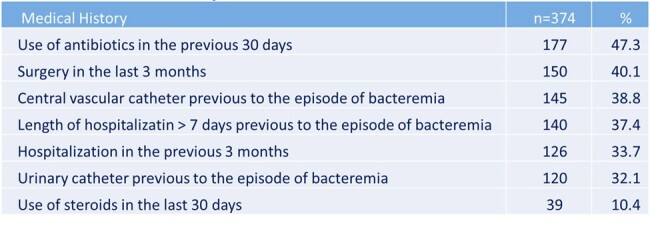
Medical History in Patients with Bacteremia

**Methods:**

This is a bidirectional, observational, analytical cohort study. Conducted with 467 patients from the Internal Medicine, Surgery, Orthopedics, Burn Unit, and Intensive Care Unit services of the HFVP who presented bacteremia between January 2021 to December 2023. After filtering the data, the patients included for analysis were 374. Different variables were analyzed, and mortality was analyzed with some factors including SOFA, NEWS2, and Pitt scores.

**Table 2.**
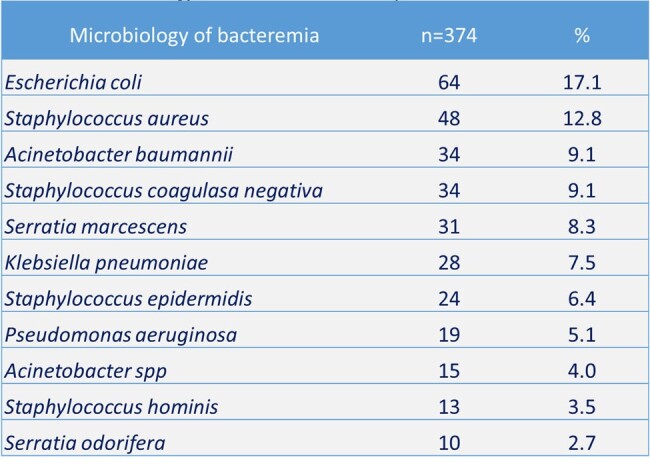
Microbiology of Bacteremia in Hospital Dr. Fernando Vélez Paiz

**Results:**

The mean age of the patients was 50.2 ± 19.9 years, and 63.7% had a comorbidity. The most frequent comorbidities were hypertension (54%), diabetes (45.1%), and chronic kidney disease (15.7%). Most of the patients were from the surgery (31.8%), internal medicine (31.8%), and intensive care unit (28.6%) departments. In the medical history were relevant factors: use of antibiotics in the last 30 days (47.3%), surgery in the last 3 months (40.1%), presence or history of central vascular catheter (38.8%) (Table 1). The most common sites of infection that were documented were respiratory (29.9%), intra-abdominal (27.8%), and urinary (22.5%). At the time of bacteremia, patients had a mean of leukocytes/mm^3^ of 15,590 ± 9,540, C-reactive protein (mg/L) of 123.0 ± 65.8, and procalcitonin (ng/mL) of 16.6 ± 19.2. The most frequent bacteria were *E. coli* (17.1%), *S. aureus* (12.8%), and *A. baumannii* (9.1%) (Table 2). 62.6% of Gram-negative were multidrug-resistant. Factors associated with mortality were recent steroid use, associated chronic disease, and multidrug-resistance (Table 3). SOFA, NEWS2, and Pitt scores correlated with the probability of survival (Figure 1).

**Table 3.**
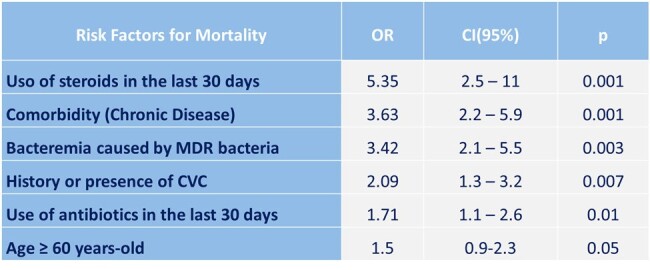
Risk Factors for Mortality in Patients with Bacteremia.

**Conclusion:**

This is the first study to characterize bacteremias in Nicaragua, finding independent risk factors for mortality and validating severity scales to predict probability of survival.

**Figure 1.**
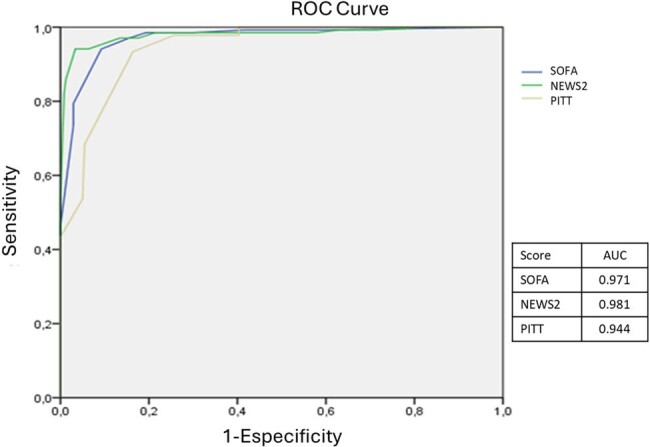
ROC Curve for SOFA, NEWS2, and PITT Score for Mortality Prediction in Patients with Bacteremia

**Disclosures:**

**All Authors**: No reported disclosures

